# Supplementary material for: Factors influencing taxonomic unevenness in scientific research: a mixed-methods case study of non-human primate genomic sequence data generation
Source: R Soc Open Sci. 2020 Sep 30;7(9):201206. doi: 10.1098/rsos.201206 (PMC7540799; doi:10.1098/rsos.201206)
Supplement: Supplementary Figures and Tables [file rsos201206supp1.pdf]

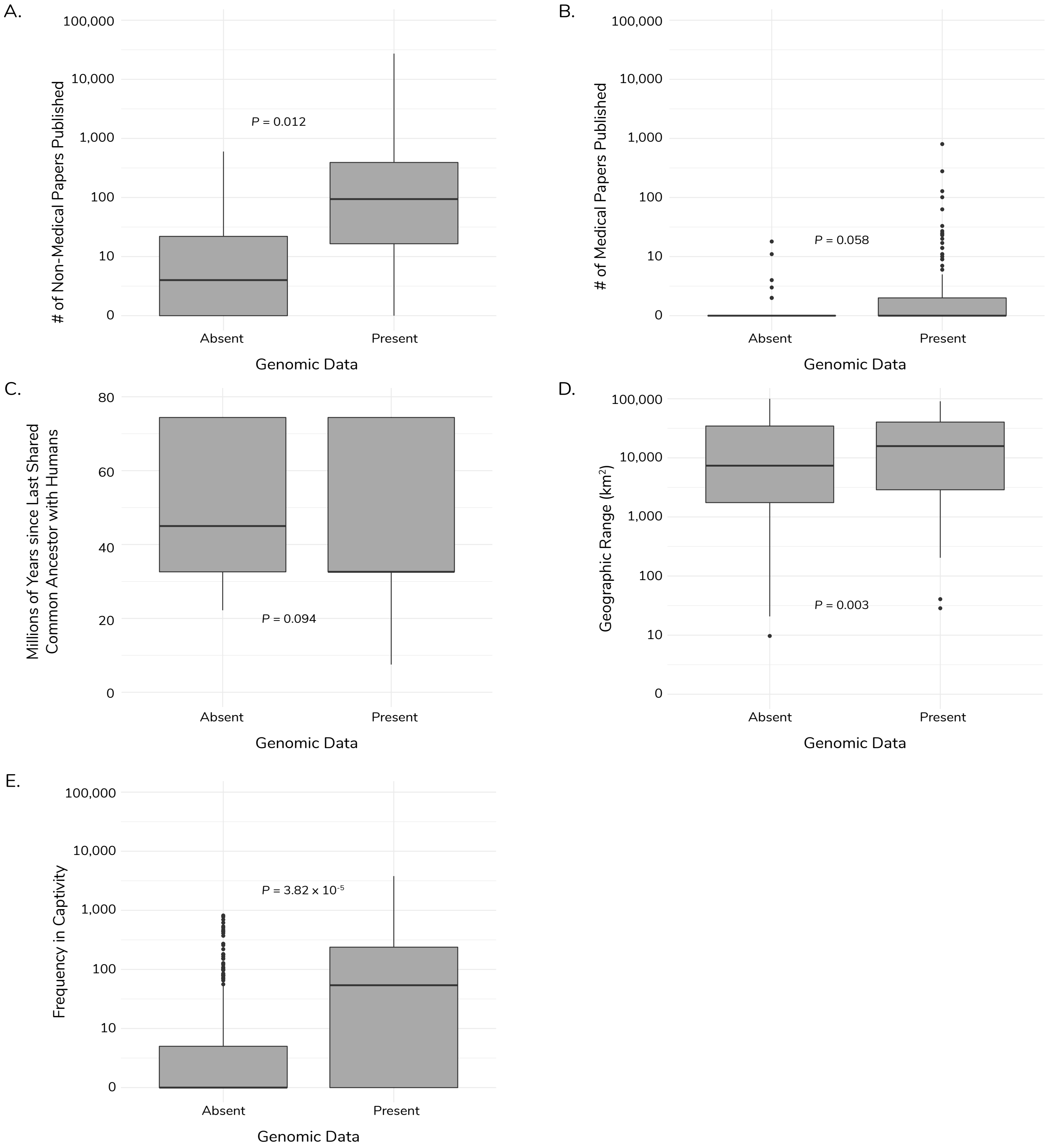

Supplementary Figure 1. Boxplots comparing species with and without any genomic data for each tested variable. The box extends from the first to the third quantile. The horizontal line within each boxplot represents the median value. The whiskers represent the lower and upper extreme value limits. The black dots represent outliers.

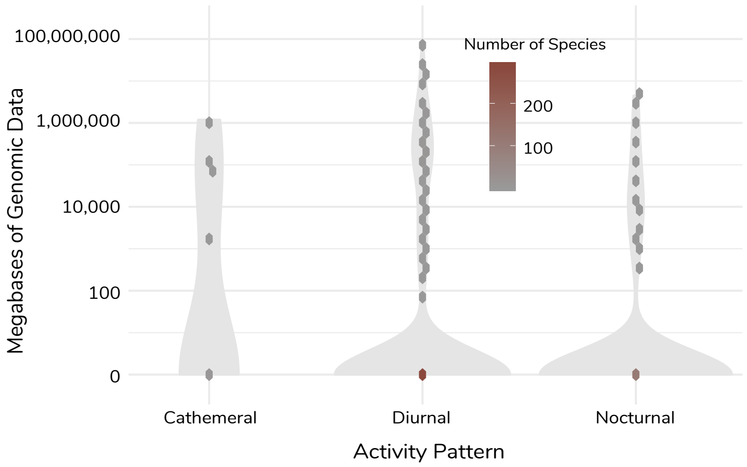

Supplementary Figure 2. Violin plots of per-species genomic data by activity pattern. Violin plot width corresponds to the density of species, which is also depicted via heatmap. There are 7 species that are cathemeral with no genomic data available, 290 species that are diurnal with no genomic data available, and 109 species that are nocturnal with no genomic data available. There are 11 species that are not represented because they did not have activity pattern information available.

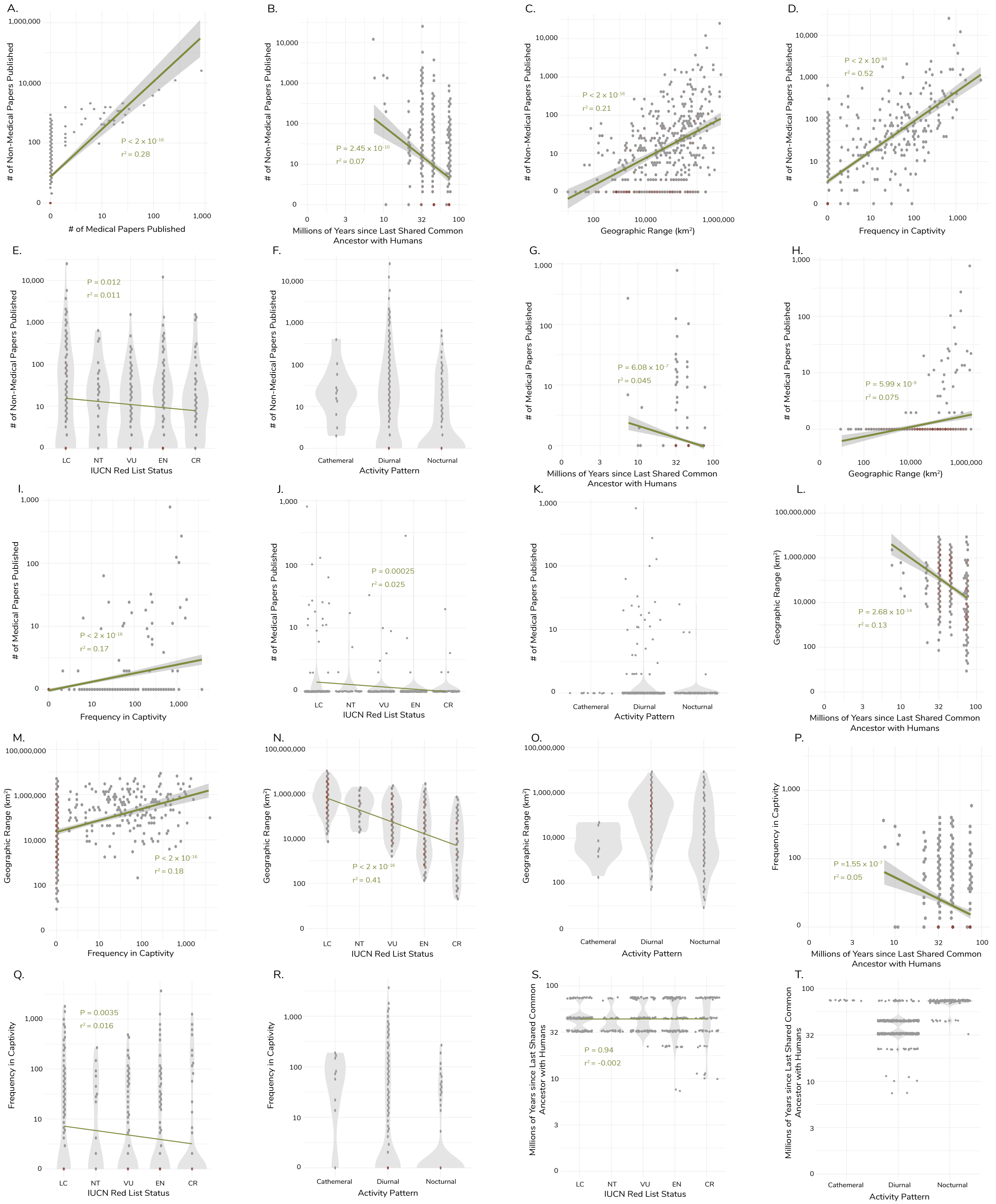

Supplementary Figure 3. Linear regressions for all predictor variables. Plots showing the relationships between the different predictor variables used within our study. We show the linear regressions, including P and r^2 values, for each comparison in green where relevant. All plots, except for J, K, S, and T, show the density of species that occupy the space on the graphs. Darker red indicates more species, while light gray indicates less species. Plots J, K, S, and T show this through the jitter of points on the graph.

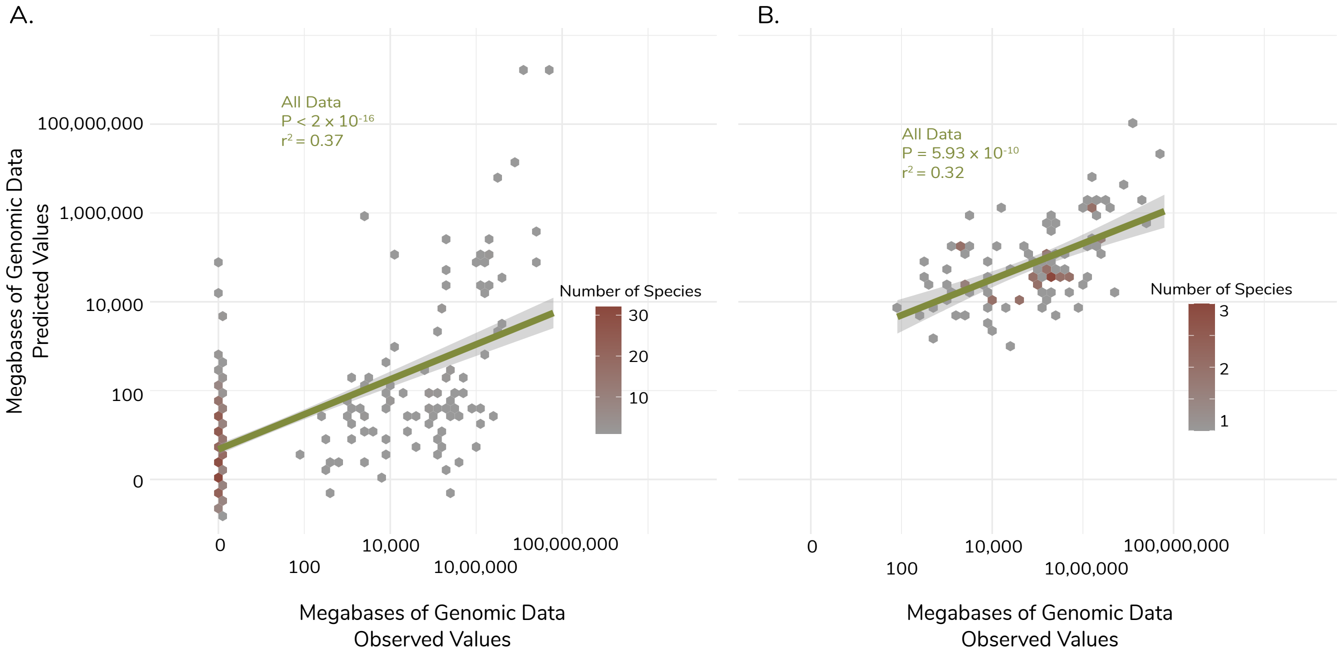

Supplementary Figure 4. Leave-one-out cross validation test. In order to test the strength of our GLMs, we performed a leave-one-out cross validation test for the entire dataset (A) and the subset of species that have genomic data present (B). To do this test, we iteratively left one sample out of the dataset and ran the GLM model. We then used the model to predict the megabases of genomic for the species that was left out. We ran models omitting each species in the dataset and plotted the observed values of megabases of genomic data (from our dataset) versus the predicted values (from the model) for each species. We then performed linear regressions on the observed versus the predicted values to see the strength of the models, reported in green.

Genus Grouping	Collapsed Genera
Callicebus	Callicebus, Plecturocebus
Callithrix	Callithrix, Cebuella, Mico
Cebus	Cebus, Sapajus
Cercopithecus	Cercopithecus, Chlorocebus, Erythrocebus, Allochrocebus
Galago	Galago, Galagoides, Otolemur, Schuirocheirus
Lagothrix	Lagothrix, Oreonax
Papio	Papio, Rungwecebus
Saguinus	Saguinus, Leontocebus
Trachypithecus	Trachypithecus, Semnopithecus

Supplementary Table 1. List of paraphyletic generic names found in genomic databases that were collapsed into a single genus for construction of Figure 1. The single genus names listed in the first column were used in Figure 1 (denoted by asterisks in the Figure) to represent data combined for multiple genera now believed to be paraphyletic, listed in the second column.

List of Interview Questions
Q1. Why did you decide to do work on xx species? Was your lab already working on this species, or was this the first time you did a particular project on this species?
Q2. What motivated you to do this study/start working with this species?
Q3. Were there factors that made studying your selected primate species easier than it would have been to study other potential species? If so, what were they?
Q4. Were there factors that made studying your selected primate species more difficult than it would have been to study other potential species? If so, what were they?
Q5. Did you face any additional challenges in working with your selected species?
Q6. Did you want to say anything additional that you didn't get the opportunity to say already?

Supplementary Table 2. List of interview questions. Interview questions were asked in the order presented in this table. At times, additional probing questions were asked regarding the answers provided for each question. However, overall, each interview followed the structure presented here.

Deposit Name	Total Data (Mb)
Cebus sp.	402
Gorilla	210,516
Macaca	422,040
Microcebus	586
Papio anubis x Papio hamadryas	35,584
Papio anubis x Papio cynocephalus	898
Papio anubis x Papio ursinus	1,162
Papio kindae x Papio cynocephalus	1,932
Papio kindae x Papio ursinus	17,119
Primates	1,176
Rhinopithecus	480,606
Unidentified	242
Unidentified monkey	9,546
**Total**	1,181,809

Supplementary Table 3. SRA deposits omitted from genomic dataset. A complete list of the SRA deposits that were removed from the dataset (see Methods), including the total amount of genomic data under each deposit name. Deposits that were not identified at the species level or that came from known species-hybrid individuals were omitted.

Data	Test	Distribution	Variable(s)[1]	AIC	p-value	r^2
Whole dataset	Linear regression	Gaussian	Non-medical publications	N/A	$<2.2 \times 10^{-16}$	0.33
Species with genomic data	Linear regression	Gaussian	Non-medical publications	N/A	6.44×10^{-12}	0.37
Whole dataset	Linear regression	Gaussian	Medical Publications	N/A	$<2.2 \times 10^{-16}$	0.27
Species with genomic data	Linear regression	Gaussian	Medical Publications	N/A	9.27×10^{-9}	0.27
Whole dataset	Linear regression	Gaussian	Frequency in zoos	N/A	$<2.2 \times 10^{-16}$	0.22
Species with genomic data	Linear regression	Gaussian	Frequency in zoos	N/A	0.000222	0.12
Whole dataset	Linear regression	Gaussian	Relatedness to humans	N/A	5×10^{-6}	0.038
Species with genomic data	Linear regression	Gaussian	Relatedness to humans	N/A	0.00106	0.092
Whole dataset	Linear regression	Gaussian	Geographical range	N/A	0.00032	0.028
Species with genomic data	Linear regression	Gaussian	Geographical range	N/A	0.0012	0.094
Whole dataset	Linear regression	Gaussian	IUCN Red List status	N/A	0.926	0.0021
Species with genomic data	Linear regression	Gaussian	IUCN Red List status	N/A	0.361	0.0016
Whole dataset	Logistic regression	Binomial	Non-medical publications + Relatedness to humans + Medical papers published + Geographical range + Frequency in zoos + IUCN Red List Status + Activity pattern	346.3	N/A	N/A
Whole dataset	GLM	Gaussian	Non-medical publications + Relatedness to humans + Medical papers published + Geographical range + Frequency in zoos + IUCN Red List Status + Activity pattern	1659.3	$<2.2 \times 10^{-16}$	0.40
Species with genomic data	GLM	Gaussian	Non-medical publications + Relatedness to humans + Medical papers published + Geographical range + Frequency in zoos + IUCN Red List Status + Activity pattern	293.26	6.16×10^{-8}	0.40

Note:
[1] "+" indicates the function used for the variables within each model.

Supplementary Table 4. Analytical models performed. A list of all models used within the study, including data used, test performed, distribution of data, variables included, AIC values, p-values, and r^2 values where applicable.
